# Supplementary material for: Imaging and identification of single nanoplastic particles and agglomerates
Source: Sci Rep. 2023 Jun 24;13:10275. doi: 10.1038/s41598-023-37290-y (PMC10290666; doi:10.1038/s41598-023-37290-y)
Supplement: Supplementary file 1 — Supplementary Information. [file 41598_2023_37290_MOESM1_ESM.pdf]

Supplementary Information for Imaging and Identification of Single Nanoplastic Particles and Agglomerates

Ambika Shorny, Fritz Steiner, Helmut Hörner, and Sarah M. Skoff*

Atominstitut, Technische Universität Wien, Stadionallee 2, Vienna, 1020, Austria

E-mail: sarah.skoff@tuwien.ac.at

This PDF file includes:

- Additional experimental results and discussion
- Figures S1, S2

Additional experimental results and discussion

Reference Measurements on a glass substrate

To be able to quantify the advantage of the surface-enhanced Raman image, we have taken micro-Raman images on a glass substrate as reference measurements. Whereas in the main part of the manuscript scans over a smaller area displaying single PS particles of different sizes are shown, Fig. S1 shows a 100×100 μm image of 800 nm PS particles on a glass substrate obtained by (a) collecting the backscattered laser light and (b) collecting the Raman scattering of the most dominant C-C stretch mode at 1002 cm^{-1}. In both images we can clearly distinguish single particles from agglomerates and find that agglomerates can

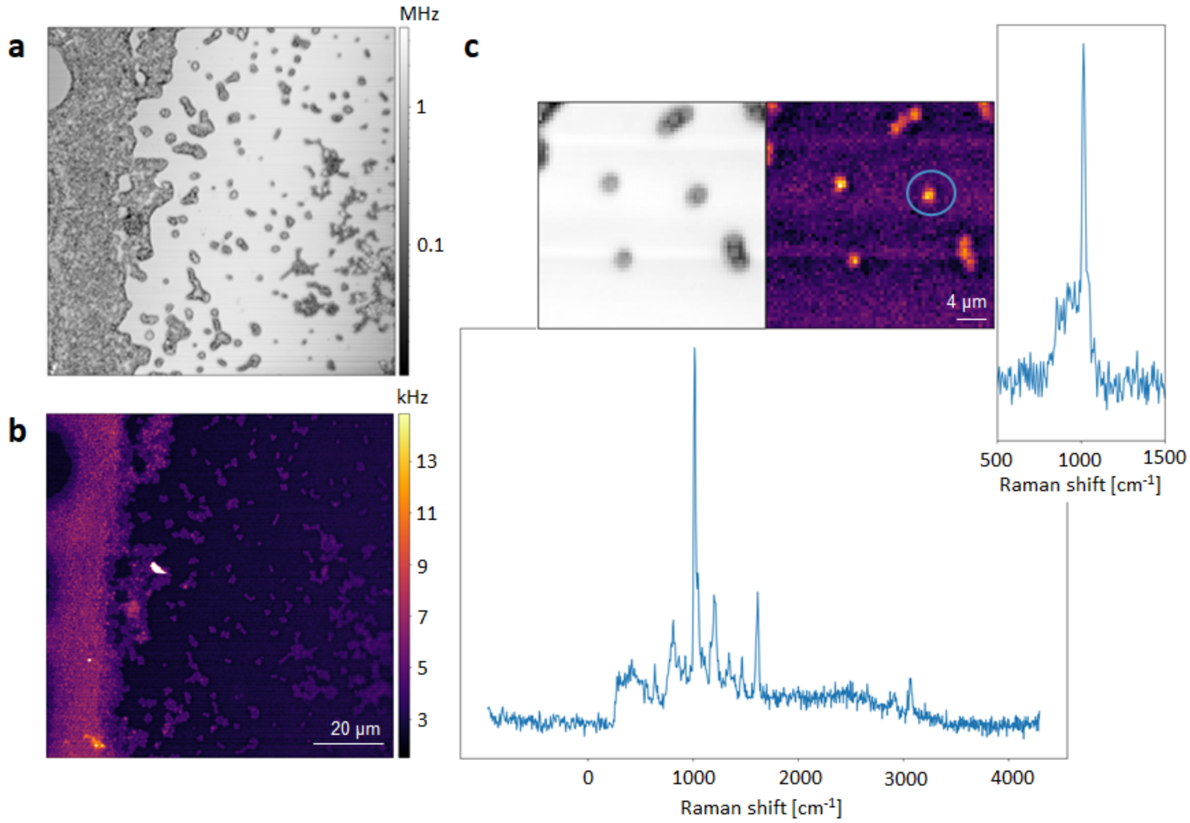

Figure S1: (a) and (b) show confocal images of 800 nm polystyrene particles on glass. In (a) the backscattered laser light is collected and in (b) the scattered Raman signal is detected. (c) shows the characteristic Raman spectrum measured on a single polystyrene nanoparticle (circled in the above image) and the inset shows the same spectrum taken with a bandpass filter to collect only the most significant Raman peak.

lead to higher count rates compared to single particles even for non-SERS substrates. Such a clear direct measurement of single particles and agglomerates even on non-SERS substrates without any post-processing of the images is already beyond the state of the art.[1–3] In Fig. S1 (c), the Raman spectrum of a single 800 nm PS particle is displayed, showing all the expected Raman peaks even without any post-processing or background subtraction. The part of the spectrum collected for creating the fast Raman images is displayed in the inset of S1 (c).

Fast Raman images

As we create our Raman images by filtering of the dominant Raman mode rather than Raman mapping, the timescale of the scans is not limited by the integration time of a spectrometer. We can thus scan the piezo faster while collecting the light with the SPCMs. Lowering the integration time in this way means that the total number of counts that are collected per pixel decreases and hence the observed contrast depends on the scattering rate. Fig. S2 shows an image of 800 nm PS spheres on the glass and the SERS substrate with an integration time of 10 ms/pixel. While on the glass, the particles in the Raman image are barely visible, the high Raman scattering count rate on the SERS subtrate ensures a strong contrast between the Raman signal and the background. For the SERS substrate in principle an integration time of even < 1 ms is feasible considering the scattering rate which is on the MHz level. In our case we are only limited to 10 ms, by the way the piezo is currently implemented in our experimental control program. For the results shown here, the most

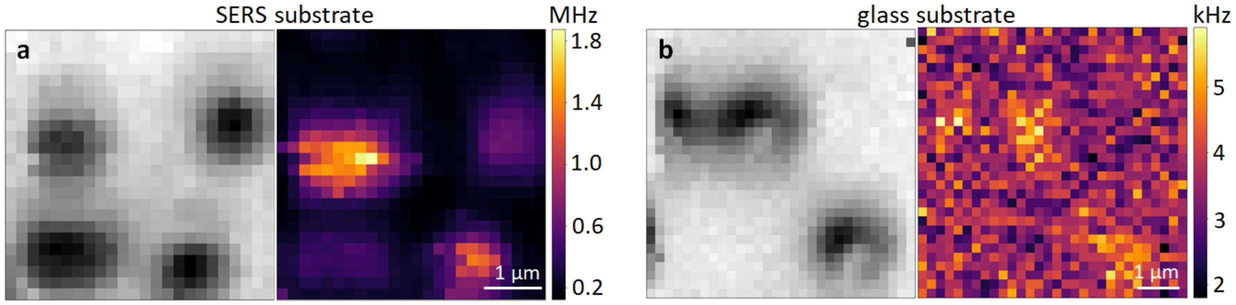

Figure S2: Fast scan of 800 nm sized particles on (a) a SERS substrate and (b) a glass substrate, with an integration time of 10 ms/pixel (only limited by piezo).

dominant Raman mode is filtered out, which already gives good results. In many Raman mapping schemes especially for low concentrations and not even single particles,[2,4–6] also only the most dominant mode can be detected and hence our method is equivalent in detection precision while being much faster.[7] In addition more modes can be filtered simultaneously by also filtering the second beam that is currently used for detection of the backscattered laser light. The two simultaneously taken images can then be correlated and thus the precision is

enhanced without the need to increase the detection time of ≤ 1 ms/pixel.

References

(1) Xu, G.; Cheng, H.; Jones, R.; Feng, Y.; Gong, K.; Li, K.; Fang, X.; Tahir, M. A.; Valev, V. K.; Zhang, L. Surface-Enhanced Raman Spectroscopy Facilitates the Detection of Microplastics <1 μm in the Environment. *Environmental Science & Technology* **2020**, *54*, 15594–15603, Publisher: American Chemical Society.

(2) Yang, Q.; Zhang, S.; Su, J.; Li, S.; Lv, X.; Chen, J.; Lai, Y.; Zhan, J. Identification of Trace Polystyrene Nanoplastics Down to 50 nm by the Hyphenated Method of Filtration and Surface-Enhanced Raman Spectroscopy Based on Silver Nanowire Membranes. *Environmental Science & Technology* **2022**, Publisher: American Chemical Society.

(3) Zhou, X.-X.; Liu, R.; Hao, L.-T.; Liu, J.-F. Identification of polystyrene nanoplastics using surface enhanced Raman spectroscopy. *Talanta* **2021**, *221*, 121552.

(4) Jeon, Y.; Kim, D.; Kwon, G.; Lee, K.; Oh, C.-S.; Kim, U.-J.; You, J. Detection of nanoplastics based on surface-enhanced Raman scattering with silver nanowire arrays on regenerated cellulose films. *Carbohydrate Polymers* **2021**, *272*, 118470.

(5) Lê, Q. T.; Ly, N. H.; Kim, M.-K.; Lim, S. H.; Son, S. J.; Zoh, K.-D.; Joo, S.-W. Nanostructured Raman substrates for the sensitive detection of submicrometer-sized plastic pollutants in water. *Journal of Hazardous Materials* **2021**, *402*, 123499.

(6) Hu, R.; Zhang, K.; Wang, W.; Wei, L.; Lai, Y. Quantitative and sensitive analysis of polystyrene nanoplastics down to 50 nm by surface-enhanced Raman spectroscopy in water. *Journal of Hazardous Materials* **2022**, *429*, 128388.

(7) Sobhani, Z.; Zhang, X.; Gibson, C.; Naidu, R.; Megharaj, M.; Fang, C. Identification

and visualisation of microplastics/nanoplastics by Raman imaging (i): Down to 100 nm. *Water Research* **2020**, *174*, 115658.
